# Angiogenic, hyperpermeability and vasodilator network in utero-placental units along pregnancy in the guinea-pig (Cavia porcellus)

**DOI:** 10.1186/1477-7827-6-13

**Published:** 2008-03-27

**Authors:** Gloria Valdés, Rafaela Erices, Cecilia Chacón, Jenny Corthorn

**Affiliations:** 1 Departamento de Nefrología, Escuela de Medicina, Pontificia Universidad Católica de Chile, Marcoleta 391, 8330024 Santiago, Chile; 2 Centro de Investigaciones Médicas, Escuela de Medicina, Pontificia Universidad Católica de Chile, Santiago, Chile

## Abstract

**Background:**

The angiogenic and invasive properties of the cytotrophoblast are crucial to provide an adequate area for feto-maternal exchange. The present study aimed at identifying the localization of interrelated angiogenic, hyperpermeability and vasodilator factors in the feto-maternal interface in pregnant guinea-pigs.

**Methods:**

Utero-placental units were collected from early to term pregnancy. VEGF, Flt-1, KDR, B2R and eNOS were analyzed by immunohistochemistry, and the intensity of the signals in placenta and syncytial streamers was digitally analysed. Flt1 and eNOS content of placental homogenates was determined by western blotting. Statistical analysis used one-way analysis of variance and Tukey's Multiple Comparison post-hoc test.

**Results:**

In the subplacenta, placental interlobium and labyrinth VEGF, Flt-1, KDR, B2R and eNOS were expressed in all stages of pregnancy. Syncytial streamers in all stages of gestation, and cytotrophoblasts surrounding myometrial arteries in early and mid pregnancy – and replacing the smooth muscle at term – displayed immunoreactivity for VEGF, Flt-1, KDR, eNOS and B2R. In partly disrupted mesometrial arteries in late pregnancy cytotrophoblasts and endothelial cells expressed VEGF, Flt-1, KDR, B2R and eNOS. Sections incubated in absence of the first antibody, or in presence of rabbit IgG fraction and mouse IgG serum, yielded no staining. According to the digital analysis, Flt-1 increased in the placental interlobium in days 40 and 60 as compared to day 20 (P = 0.016), and in the labyrinth in day 60 as compared to days 20 and 40 (P = 0.026), while the signals for VEGF, KDR, B2R, and eNOS showed no variations along pregnancy. In syncytial streamers the intensity of VEGF immunoreactivity was increased in day 40 in comparison to day 20 (P = 0.027), while that of B2R decreased in days 40 and 60 as compared to day 20 (P = 0.011); VEGF, Flt-1, KDR, B2R and eNOS expression showed no variations. Western blots for eNOS and Flt-1 in placental homogenates showed no significant temporal differences along pregnancy.

**Conclusion:**

The demonstration of different angiogenic, hyperpermeability and vasodilator factors in the same cellular protagonists of angiogenesis and invasion in the pregnant guinea-pig, supports the presence of a functional network, and strengthens the argument that this species provides an adequate model to understand human pregnancy.

## Background

The successful evolution of pregnancy requires finely tuned adaptations to permit an adequate exchange between fetal and maternal blood. On one hand, the fetal and maternal circulations establish close contact in the progressively extended and thin vascular structure of the placenta. On the other, utero-placental blood flow increases progressively, by transformation of the uterine arteries into large bore non-reactive vessels, achieved by cytotrophoblasts that disrupt the smooth muscle of the uterine arteries and replace their endothelium. Thus, both the angiogenic and invasive properties of the cytotrophoblast are critical in determining the fate of pregnancy [1-6].

Vascular endothelial growth factor (VEGF) seems to be fundamental to placentation. It is expressed in the utero-placental interface of several species [7-13], in humans localizes mainly in the invading front of anchoring columns and in endovascular cytotrophoblasts, is down regulated in preeclampsia, and the blockade of its binding decreases cytotrophoblast expression of integrin α1, and increases apoptosis [14]; its removal from culture medium induces massive apoptosis of uterine microvascular endothelial cells [15]. Moreover, VEGF receptor knockout mice have a high embryonic lethality associated to defective angiogenesis [16,17]. VEGF could also increase vascular permeability in the endometrium, as demonstrated for tumoral cells [18]. Later, localized in perivascular trophoblasts, could through its vasodilator effect prime the uterine arteries for invasion, as has been suggested for NO [19], enhancing their high blood flow.

**Figure 1:**
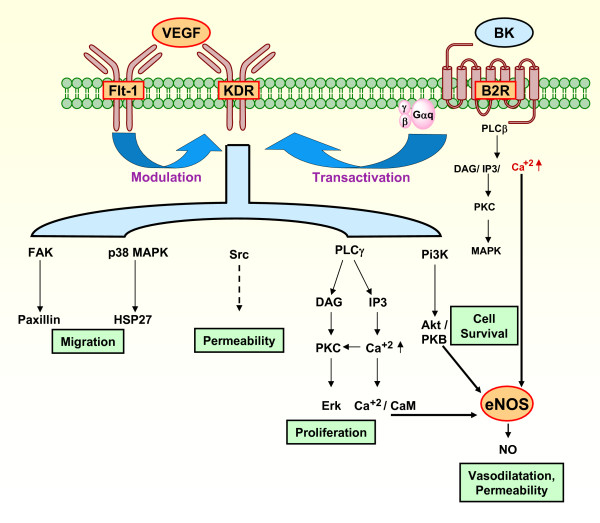
**Signaling pathways that contribute to VEGF induced angiogenesis, and a proposal for their participation in the development of the utero-placental interface by participating in proliferative, invasive, vasodilatory and permeability changes essential for cell invasion and angiogenesis.** VEGF activates eNOS through pathways including Akt/PKB, Ca^+2^/CaM, and PKC [20-24]. Flt-1 may negatively regulate KDR, but might also promote its activity [20]. Bradykinin stimulates eNOS through Ca^+2^, induces EC to form tubes, and transactivates the KDR [26]. The factors studied have been depicted in orange areas surrounded by a red border, known mechanisms of downstream activation by interrupted arrows, and unknown mechanism of downstream activation by interrupted arrow. VEGF, vascular endothelial growth factor; BK, bradykinin; eNOS, endothelial nitric oxide synthase; PLC β, phospholipase C -β; PLCγ, phospholipase C -γ; DAG, diacylglycerol; IP3, inositol (1,4,5)-triphosphate; PKC, protein kinase C; MAPK, mitogen-activated protein kinase; FAK, focal adhesion kinase; PI3K, phosphoinositide 3-kinase; p38 MAPK, p38 mitogen-activated protein; Erk, extracellular regulated kinase; HSP27, heat protein; CaM, calmodulin; EC, endothelial cell.

The pluripotential effects of VEGF are exerted by the activation of its two receptors, Flt-1 and KDR [20,21]. Of these, KDR can be the sole mediator of the physiological and pathological effects of VEGF, being capable of promoting angiogenesis and hyperpermeability in vivo. Flt-1 modulates KDR, preventing the excessive and disorganized formation of endothelial cells, promotes migration of endothelial cells, and upregulates the expression of MMP-9 [22]. In humans VEGF receptors are expressed in anchoring columns, are upregulated within their first cell layers and downregulated in severe preeclampsia and in the hemolysis, elevated liver enzymes and low platelet (HELLP) syndrome. *In vitro *inhibition of ligand binding to these receptors decreases cytotrophoblast invasion [14]. Both Flt-1 and KDR trigger a cascade that activates eNOS, via phosphatidilinositol 3-kinase and phospholipase Cγ1 respectively [20,23,24] (Figure 1). The release of NO, considered a second messenger for VEGF-driven angiogenesis with its consequent vasodilation, represent initiating events in sprouting angiogenesis [21].

Bradykinin is also a potent stimulus of eNOS, and NO production, through Ca ^2+ ^mediated mechanisms. Depending on the cell types, it can induce proliferative or antiproliferative effects, and appears to be an important agent in ischemia-induced angiogenesis [25]. The two cognate receptors of the kallikrein-kinin system are the type-1 and type-2 receptors (B1R and B2R), being the B2R ubiquitous and the main receptor of the classical effects of kinins (vasodilation, hyperpermeability), in contrast to the B1R, which is involved in tissue injury, reaction to cytokines or microbial products. The effects of VEGF are also enhanced by bradykinin [26,27].

The present study aims at demonstrating the localization and the variations in the expression of VEGF, Flt-1, KDR, B2R and eNOS along pregnancy in guinea-pigs. We hypothesize that the localization of these functionally interrelated factors supports a network of angiogenic, hyperpermeability and vasodilator factors (Fig. 1), that participates in placental angiogenesis and cytotrophoblast invasion.

The pregnant guinea-pig was used in this study as it shares with the human utero-placental interfase a series of similarities, as a hemomonochorial structure, a subplacenta that resembles the anchoring villi as a source of extravillous trophoblasts, a decidual invasion and a transformation of the uterine arteries into low resistance high flow vessels [28-30]. Apart from the morphofunctional similarities, this species helps to circumvent the ethical and technical constraints that hinder the sequential study of normal or deranged human pregnancy.

We describe the expression of VEGF, Flt-1, KDR, B2R and eNOS in the same cell types of the feto-maternal interface of the guinea-pig, the subplacenta, the interlobar and labyrinthine placenta, syncytial streamers, peri and intravascular cytotrophoblasts, and fetal and maternal endothelial cells. This study supports our hypothesis for a functional role of this angiogenic, hyperpermeability and vasodilator network.

## Methods

Guinea-pigs (Pirbright white ~600 g) were kept under controlled humidity, temperature, and a 12-hour light: dark cycle. The animals were fed with Xtravital Special Food for Cavies (Beaphar, Holland) and supplemented with ascorbic acid in the drinking water. Litter size varied from 2–5 fetuses. Females were examined daily, and when vaginal opening was observed they were caged with fertile males. The day in which a vaginal plug, or sperms were observed in vaginal smears, was defined as day one of pregnancy. The experiments were approved by the IRB, and conducted according to the *Guide for the Care and Use of Laboratory Animals *(National Research Council, USA). The animals were deeply anesthetized with a mixture of ketamine (100 mg/kg) and xylazine (4 mg/kg) given intraperitoneally. Twenty dams were sacrificed in early (15, 20 days), mid (30, 45 days) and term (60 days) pregnancy (≈ length of pregnancy in the guinea-pig 63 days). The uterus and feto-placental units were removed; in some units a central slice through the placenta, subplacenta, implantation site and underlying myometrium was fixed as a single block; in other units, the placenta, endometrium, and mesometrium were separately dissected. The animals were euthanized with an overdose of anesthetic. Tissues were fixed immediately with phosphate-buffered 4% formalin for 24 h. The fixed tissues were dehydrated in a graded series of ethanol and xylene, and embedded in Paraplast-Plus^® ^(Sigma, St. Louis, MO). Serial sections (5 μm) were mounted on silanized slides.

### Immunostaining procedure

All immunostaining procedures were performed at room temperature; deparaffinized sections were rehydrated through ethanol and methanol, rinsed three times for five minutes in PBS-50 mM Tris-HCl pH 7.8 and submitted to heat antigen retrieval using citrate buffer pH 6.0 for eNOS, VEGF, Flt-1 and KDR, cytokeratin and von Willebrand factor, and Tris-HCl-EDTA pH 9.0 for muscle actin and cytokeratin-7. Endogenous peroxidases were blocked by incubating in 10% H_2_O_2 _for 20 minutes. Sections were incubated in a humid chamber for 30 minutes with protein block (Cas-Block^®^, Zymed, San Francisco, CA) followed by incubation for 18 h at 4°C with the primary antibodies: mouse monoclonal anti-eNOS (1:50, clone 3 BD Transduction Laboratories), VEGF (1:50, clone JH 121, Upstate); rabbit polyclonal KDR (1:500, Upstate) and Flt-1 (1:1000, Santa Cruz Biotechnology, Inc). The B2R antibody (1:4000), mixture of 8 polyclonal rabbit antisera that recognizes the various domains of the receptor, was raised against the rat kinin B2R, and was kindly donated by Dr. Werner Müller-Esterl, Frankfurt Universitäts, Germany [31].

Sections were immunostained using a biotin-streptavidin-peroxidase system (DakoCytomation, Carpinteria, CA). Finally, the samples were incubated for 15 minutes with 0.1% (w/v) 3-3'-diaminobenzidine in buffer containing 0.05% H_2_O_2_. The slides were counterstained with Harris hematoxylin (Sigma, St. Louis, MO).

Cytotrophoblasts were identified by staining with an anti-pancytokeratin mouse monoclonal antibody (1:100, P2871, Sigma, St. Louis, MO), and in addition by cytokeratin 7-specific antibody (1:10, Clone OV-TL/12/30 DakoCytomation) that binds to all trophoblast populations and epithelial cells, and displays no reactivity for mesenchymal cells [32]. Smooth muscle and endothelial cells were characterized with antibodies against muscle actin (clone HHF35, 1:1000) and von Willebrand factor (A0082, 1:400) both obtained from DakoCytomation. The specificity of the staining was determined by incubation of sections in the absence of the first antibody, or in the presence of rabbit IgG fraction (1:100 and 1:4000) and mouse IgG serum (1:50), both from Dako Cytomation.

### Digital image analysis

The immunostained sections of the placenta and endometrium were photographed with a Nikon CoolPix 4500 camera (Nikon Inc., Tokyo, Japan) coupled to a Zeiss AxioImager AX.10 microscope (Carl Zeiss, CA) using a 20× and 40× objectives [33].

The immunohistochemical expression of VEGF, Ft1-1, KDR, eNOS and B2R was digitally analyzed in a total of fifteen fields from each placenta and endometrium, which were photographed from five sections per dam, in three animals in each of the following periods: early (20 days), mid (40 days) and term (60 days) pregnancy. The images were loaded into the ImageJ v.1.34 software (National Institutes of Health, Bethesda, MD) for analysis. A total of 243 syncytial streamers were manually delineated, extracted from the surrounding tissue to a separate image file and saved in JPEG format. Immunoreactive signals were extracted from the images with a color deconvolution algorithm [34], integrated in the ImageJ software. After conversion of pixel luminosity values to an optical density scale, the integrated optical density was measured in the previously extracted positive staining images and normalized by the positive area in each microphotograph. The signal intensity (I) was calculated as I = 10·∑OD/A in dB/μm^2^, being ∑OD the integrated optical density, and A the area of positive staining (μm^2^) [35].

### Protein extraction

Total proteins from placenta from early, mid and term pregnancy were extracted using 5 ml/g of 20 mM Tris-HCl buffer containing 10 mM EDTA, 2 mM phenylmethylsulfonylfluoride, 5 μM leupeptin, 50 μg/ml soybean trypsin inhibitor, 0.05% Brij-35 and 0.02% NaN_3 _at pH 7.4. Tissues were homogenized with a Tekmar Tissumizer (Cincinnati, OH) for one minute on ice. Crude homogenates were centrifuged at 4000 rpm for 20 minutes, then 0.2% SDS was added to the supernatant, kept for 1 hour at 4°C, centrifuged at 14000 rpm for 20 min at 4°C and finally stored at -70°C. Protein content was determined according to the Lowry method [36].

### Western blot analysis

Equal amounts of protein (100 μg/lane) were separated using 10% SDS-PAGE under reducing conditions and transferred to nitrocellulose membranes (Biorad, Hercules, CA), blocked with 3% nonfat dry milk in PBS-0.1% Tween-20 buffer (PBS-T) and incubated overnight at 4°C with the same primary antibodies used in immunohistochemistry: mouse monoclonal anti-eNOS (1: 1000, clone 3 BD Transduction Laboratories) and rabbit polyclonal Flt-1 (1: 200, Santa Cruz Biotechnology, Inc) diluted in blocking buffer.

The membranes were washed three times for five minutes in PBS-T buffer, incubated with HRP-conjugated anti-mouse or anti-rabbit secondary antibodies (both 1:3000, Biorad, Hercules, CA) for one hour at room temperature and developed with chemiluminescence reagent (NEL-103, Western Lighting, Perkin-Elmer, MA) [36,37]. Membranes were exposed to CL-xPosure film (Pierce, Rockford, IL). Equal protein loading (100 ug/lane) was confirmed with Ponceau-S red staining (Sigma, St. Louis, MO). Images were scanned at 16-bit/600 dpi resolution with an Epson Perfection 3490 scanner (Epson Corporation, CA), saved as tiff files and calibrated to an optical density scale. The integrated optical density of bands was quantitated using the ImageJ v.1.34 software.

Purified human Flt-1 and eNOS yielded bands with approximate molecular weights of 180 and 140 kDa respectively [38,39].

### Statistical analysis

Overall differences between the three studied periods of pregnancy were assessed with SPSS v 10 (SPSS Inc., Chicago, IL) using the one-way analysis of variance and Tukey's Multiple Comparison post-hoc test. P < 0.05 was chosen as significance level for all analyses performed. Results are expressed as means ± SEM.

## Results

Spatio-temporal expression of VEGF, Flt-1, KDR, B2R and eNOS in utero-placental units.

### Subplacenta

**Figure 2:**
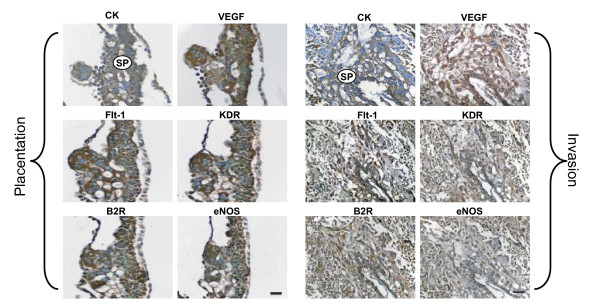
**In subsequent sections of a utero-placental unit obtained in day 15 of pregnancy, the multilayered subplacenta (SP) gave rise to the placental sprouts, and to the syncytial streamers that penetrate the adjacent decidua.** The subplacenta, the placental sprouts and the syncytial streamers expressed VEGF, Flt-1, KDR, B2R and eNOS. Cytotrophoblasts were characterized by cytokeratin (CK) staining. Bar = 100 μm.

The plied multilayered subplacenta represented the main, if not the exclusive, source of trophoblasts, as syncytial sprouts could be observed as early as day 15 of gestation emerging from its abluminal side to penetrate the first inner third of the endometrium. In addition, syncytiotrophoblast buds, emerging from the luminal side, constituted the earliest expression of the subplacenta. At this early stage, and throughout pregnancy, the subplacental syncytiotrophoblasts expressed granular immunoreactivity for VEGF, Flt-1, KDR, B2R and eNOS (Fig. 2).

### Placenta

**Figure 3:**
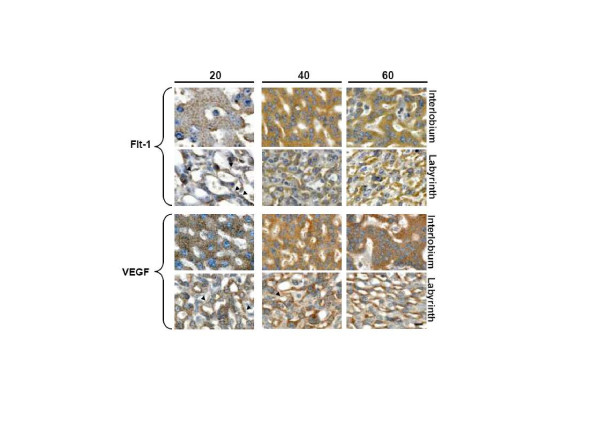
**Syncytiotrophoblasts composing the interlobium and the labyrinth expressed Flt-1 and VEGF, in sections obtained in days 20, 40 and 60 of pregnancy at a high magnification (1000×).** The immunoreactivity showed a granular pattern for interlobar Flt-1, and for interlobar and labyrinthine VEGF; labyrinthine Flt-1 displayed a diffuse cytoplasmic staining. Arrowheads highlight linear signal in endothelial cells. Bar = 100 μm.

From day 15 of pregnancy to term trabeculae and syncytiotrophoblast plates, originating from the subplacenta, expressed immunoreactivity for VEGF, Flt-1, KDR, B2R and eNOS, as granules in the thick bands of syncytiotrophoblast of the interlobium; in the labyrinth immunoreactivity for all factors displayed a diffuse reaction in the thin syncytial trabeculae and a thin lineal one in the endothelial cells. The cellular density of the interlobium showed a mild increase as pregnancy progressed, while that of the labyrinth increased markedly between days 40 and 60 as compared to day 20 (Fig. 3).

**Figure 4:**
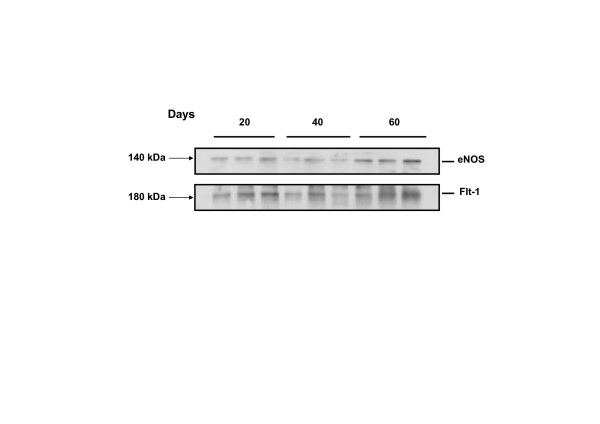
**Representative blot of placental homogenates from days 20, 40 and 60 of pregnancy for FLT-1 and eNOS.** Purified human Flt-1 and eNOS yielded bands with approximate molecular weights of 180 and 140 kDa respectively. No significant differences were observed between the means of the 3 homogenates included in each study period, using one-way analysis of variance and Tukey's Multiple Comparison post-hoc test.

The digital quantification of the reactivity showed that in the interlobium Flt-1 increased in days 40 and 60 (10.4 ± 0.62 and 10.2 ± 0,23 dB/μm^2 ^respectively), as compared to day 20 (8.3 ± 0.10 dB/μm^2^; P = 0.016), and in the labyrinth in day 60 (10.6 ± 0.32 dB/μm^2^), as compared to days 20 and 40 (7.9 ± 0.83 and 10.0 ± 0.20 dB/μm^2 ^respectively; P = 0.026). VEGF showed no variations in the interlobium or labyrinth (Fig. 3). KDR, B2R, and eNOS (not shown) displayed no changes. Western blots of placental homogenates showed no significant differences for Flt-1 and eNOS (Fig. 4).

### Endometrium

As early as day 15 of gestation, VEGF in cytokeratin positive cells was expressed in pericytes, and in invasive trophoblasts coalescing into syncytial streamers that migrated into the endometrium. Intraluminal plugs were observed in some arteries from day 20 onwards (not shown).

**Figure 5:**
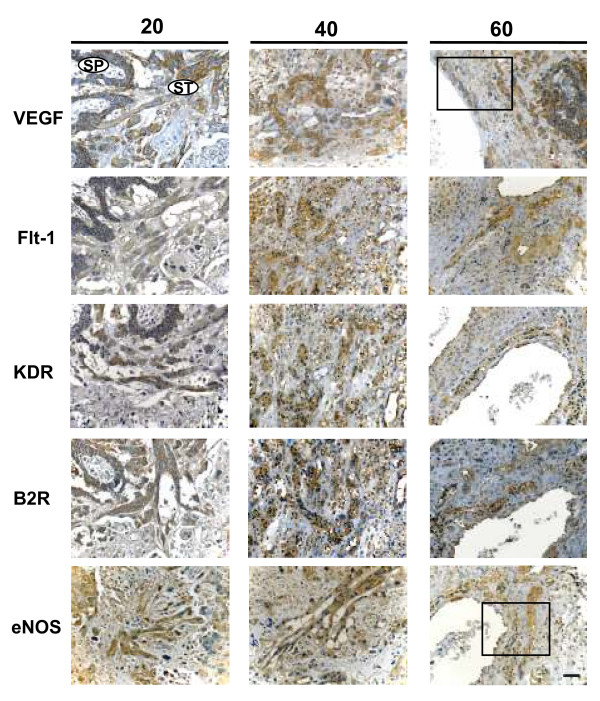
**Syncytial streamers (ST) in the subplacenta (SP) and in the decidua expressed VEGF, Flt-1, KDR, B2R and eNOS in days 20, 40 and 60 of pregnancy.** Bar = 100 μm. Rectangle defines the area shown at a higher magnification (400×) in Figure 6.

VEGF, Flt-1, KDR, B2R and eNOS immunoreactivity in syncytial streamers showed a granular staining (Fig. 5). Quantification by digital analysis showed an increased VEGF expression in day 40 (10.1 ± 0.8 dB/μm^2^) in comparison to day 20 (6.8 ± 0.7 dB/μm^2^; P = 0.027), while B2R decreased in days 40 (7.3 ± 0.4 dB/μm^2^) and 60 (7.6 ± 0.1 dB/μm^2^) as compared to day 20 (9.4 ± 0.5 dB/μm^2^; P = 0.011); no differences were observed for Flt-1, KDR and eNOS.

**Figure 6:**
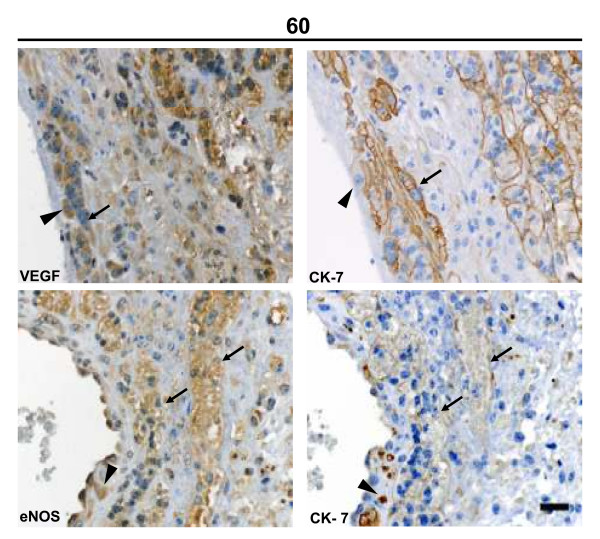
**Syncytial streamers (arrows) in the vicinity of a decidual blood vessel in late pregnancy displayed granular VEGF and eNOS reactivity, and a linear pattern for cytokeratin-7 in the syncytial membrane.** Intramural cytotrophoblasts (arrowheads) near the vessel lumen also expressed a granular eNOS reactivity, and intense intracytoplasmatic cytokeratin-7 staining. Bar = 100 μm.

Syncytial streamers were observed in the periphery of large decidual blood vessels near the subplacenta in mid and term pregnancy. In early pregnancy syncytial streamers were long thin projections of multinucleated trophoblasts. In mid pregnancy they formed thick and short aggregations, and in late pregnancy surrounded the arteries. Intramural cytotrophoblasts expressed all studied factors, as shown for VEGF and eNOS (Fig. 6).

### Myometrial and mesometrial arteries

**Figure 7:**
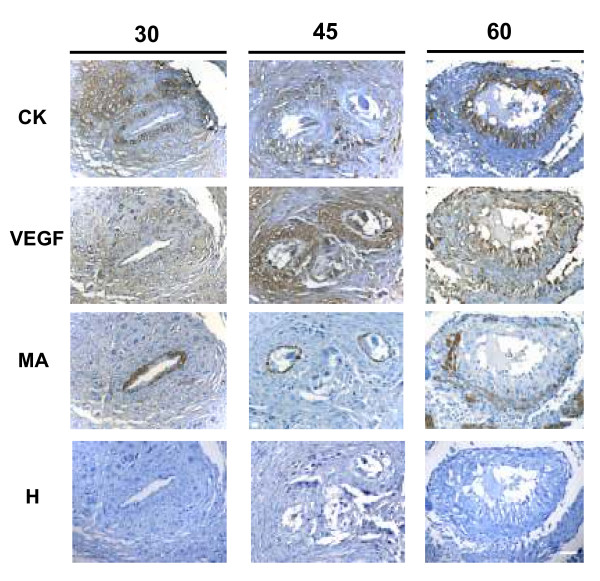
**Spiral arteries observed in sequential sections of myometrium obtained in days 30, 45 and 60 of pregnancy.** Cytokeratin positive trophoblasts expressed VEGF. The remaining vascular smooth muscle layers were positive for muscle actin (MA), and have been almost replaced in day 60. Bar = 100 μm.

Myometrial arteries showed an intact thick (30 days) or a thin, partly disrupted (45 days) vascular smooth muscle layer, and were surrounded by cytotrophoblasts expressing VEGF. VEGF was also observed in swollen endothelial cells at 45 and 60 days. In late pregnancy cytotrophoblasts almost replaced the smooth muscle layer, attained the luminal border; and presented a granular staining for VEGF (Fig. 7). Flt-1, KDR, eNOS and B2R had a similar expression than that of VEGF along the different stages of pregnancy (not shown).

**Figure 8:**
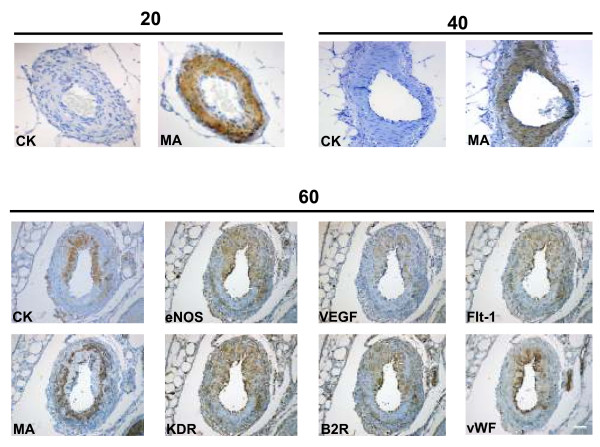
**Mesometrial arteries obtained in days 20 and 40 of pregnancy showed in sequential sections an intact smooth muscle layer characterized by muscle actin (MA), and no cytokeratin positive cells.** In day 60, intramural trophoblasts, characterized by cytokeratin (CK), expressed VEGF, Flt-1, KDR, B2R and eNOS, as did the swollen endothelial cells, identified by von Willebrand Factor (vWF). The remaining vascular smooth muscle, positive for muscle actin (MA), was disrupted. Bar = 100 μm.

**Figure 9:**
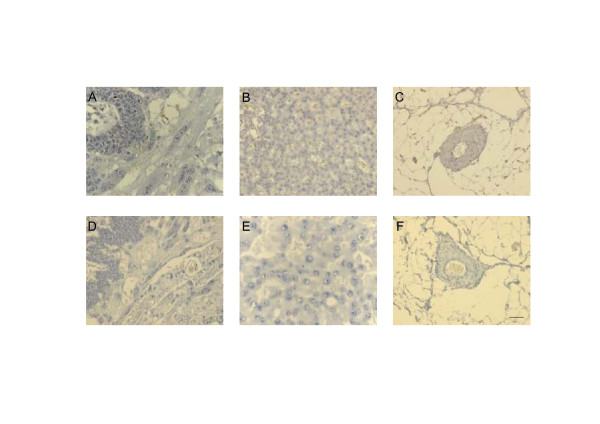
**Control sections at different days of pregnancy of the subplacenta and syncytial streamers (A, D), interlobium (B, E) and mesometrial arteries (C, F), which yielded no immunoreactivity incubated with non-specific IgG from mouse (1:50) and rabbit (1:100), species in which the antibodies were raised.** Mouse IgG serum in (A, B, C) and rabbit IgG fraction in (D, E, F). Bar = 100 μm

Mesometrial arteries in early (20 days) and mid (40 days) pregnancy had a thick multilayer of smooth muscle cells and no trophoblasts were observed in their periphery or within the muscle layer. At term pregnancy (60 days) cytotrophoblasts replaced part of the muscle layer, and presented a positive signal for VEGF, Flt-1, KDR, B2R and eNOS. Swollen endothelial cells, identified by vWF, also expressed VEGF, Flt-1, KDR, B2R and eNOS (Figure 8). Control sections incubated with rabbit and mouse non-specific immunoglobulin (Figure 9), or in absence of the first antibody (not shown), yielded no staining in different structures and stages of pregnancy.

## Discussion

The present study provides the first demonstration that VEGF, its receptors Flt-1 and KDR, eNOS and the B2R, angiogenic, vasodilatory and hyperpermeability factors, are expressed in the same cell types of the guinea-pig feto-maternal interface along pregnancy. We postulate that these functionally interrelated factors represent a network that participates in placental angiogenesis and trophoblast invasion in an autocrine/paracrine way [1].

We will now analyze the potential effect of these factors regarding the development and maintenance of the utero-placental unit. The development of the placenta from endothelial cell precursors in the first syncytial buds requires enhanced vascular permeability, a consistent feature of angiogenesis; leaky vessels extravasate fibrinogen and plasminogen, which clotting into crosslinked fibrin and plasmin respectively, could favor endothelial cell adhesion and migration, as for tumoral cells [18]. Plasmin could activate MMPs, and in addition to kallikrein also present in utero-placental units in guinea-pigs [33], could participate in basement membrane degradation. Once the placenta is developed, the anticoagulating effect of NO and bradykinin could prevent platelet aggregation in its intricate vascular spaces, favorable targets for platelet aggregation.

In the attachment and implantation stages, the increased vasodilatation of uterine blood vessels in the implantation sites probably increases blood flow to meet the demands of oxygen and nutrients of the embryo, prior to the development of the placental bed. It seems feasible that subplacental VEGF, bradykinin, and NO could generate edema, a prominent feature of decidualization, and jointly with changes in extracellular matrix could facilitate trophoblast penetration of the underlying endometrium. The subsequent propagation of syncytial streamers could convey these effects to the deeper decidua, to finally facilitate disruption of the arterial wall. In the vicinity of uterine arteries these factors could prime the maternal vessels for invasion by relaxing smooth muscle cells and increasing arterial compliance, as proposed by Nanaev and cols[19].

Our observations on the localization of eNOS extend those of Nanaev and cols. who described positive eNOS extravillous trophoblasts extending from the junctional zone into the myometrium, as well as in those surrounding mesometrial arteries and replacing endothelial cells. Our finding of VEGF, eNOS and B2R in syncytial streamers supports a similar role in priming of spiral arteries. In comparison from a recent studies of Kaufmann and cols., and Bosco and cols., we were unable to recognize the isolated invasive extravillous trophoblasts described in the transitional zone lateral to the subplacenta of the guinea-pig and degu [30,40]. These trophoblasts are reported to be cytokeratin negative while traversing the endometrium, and thus are hard to identify; we postulate that these are later represented by intramural cytotrophoblasts, which having switched their phenotype are able to express cytokeratin and the panel of factors here studied.

Several factors may be responsible for the discrepancy between the significant temporal variations observed in immunohistochemistry and the absence of changes observed in western blotting. First, the inherent differences of both techniques; while immunohistochemistry depends on the exposition of the epitopes, thus on the conformation of the protein, western blotting depends on its solubility and integrity along the stages of homogenization. However, both methods complement each other; while placental homogenates including interlobium and labyrinth demostrated proteins with the equivalent weights of Flt-1 and eNOS, the immunohistochemistry determined the protein expression in each of the different zones. Though the discrepant findings between both methods pose the need to pursue the characterization of the temporal variations, the immmunohistochemistry suggests an upregulated expression of Flt-1 in the interlobium in mid and late gestation and in the labyrinth near term, as well as of VEGF and the B2R in syncytial streamers in early and mid gestation.

In vivo and in vitro studies in the guinea-pig need to be done to evaluate the individual importance of the factors here described. The alterations induced by interfering with VEGF through the soluble form of Flt-1 (sFlt-1) [41,42], the decrease in cytotrophoblast invasion and expression of integrin α 1 [14] and the increased apoptosis by inhibition of the ligand binding of VEGF have proven the functional relevance of its pathway. The importance of the nitridergic system has been confirmed by the blockade of eNOS with L-NAME [43-45]. Alterations in pregnancy in Brown Norway rats [46], a strain with a defective kallikrein-kinin system [47], and the high predictive index of preeclampsia of low urinary kallikrein in early human pregnancy [48], suggest that a deficient kallikrein-kinin system may hinder the local adaptations of pregnancy, thus its functional role deserves to be elucidated.

In humans, immunoreactive VEGF, Flt-1, KDR [8,49] and eNOS [50-55] have been detected in villous cyto and syncytiotrophoblasts, fetal capillaries, and in extravillous trophoblasts; moreover, the B2R has been described in these cell types, and in addition in intraarterial trophoblasts [51]. The localization of these factors in placental syncytiotrophoblasts, fetal endothelial cells, and invading trophoblasts in the guinea-pig, bears a concordance with that described for cell types that in the human morphologically and functionally resemble the placental syncytium and endothelium, and the invasive trophoblasts.

## Conclusion

The expression of angiogenic, permeability enhancers and vasodilatory factors in the human and guinea-pig utero-placental interface supports for them complex, and perhaps redundant, interrelated functional roles. Moreover, this study underscores the importance of the model of the pregnant guinea-pig to understand human pregnancy.

## Competing interests

The author(s) declare that they have no competing interests.

## Authors' contributions

The study design, the histological analysis, the interpretation of the data and the drafting of the manuscript were done by JC and GV, the animals were sacrificed by JC, RE and CC; the immunohistochemistry was performed by JC, RE and CC, western blots, image and densitometrical analysis were done by RE. All authors read and approved the final manuscript.
